# Predicting proteome allocation, overflow metabolism, and metal requirements in a model acetogen

**DOI:** 10.1371/journal.pcbi.1006848

**Published:** 2019-03-07

**Authors:** Joanne K. Liu, Colton Lloyd, Mahmoud M. Al-Bassam, Ali Ebrahim, Ji-Nu Kim, Connor Olson, Alexander Aksenov, Pieter Dorrestein, Karsten Zengler

**Affiliations:** 1 Bioinformatics and Systems Biology, University of California, San Diego, La Jolla, California, United States of America; 2 Department of Bioengineering, University of California, San Diego, La Jolla, California, United States of America; 3 Department of Pediatrics, University of California, San Diego, La Jolla, California, United States of America; 4 Collaborative Mass Spectrometry Innovation Center, Skaggs School of Pharmacy and Pharmaceutical Sciences, University of California San Diego, San Diego, California, United States of America; 5 Center for Microbiome Innovation, University of California, San Diego, La Jolla, California, United States of America; Argonne National Labs, UNITED STATES

## Abstract

The unique capability of acetogens to ferment a broad range of substrates renders them ideal candidates for the biotechnological production of commodity chemicals. In particular the ability to grow with H_2_:CO_2_ or syngas (a mixture of H_2_/CO/CO_2_) makes these microorganisms ideal chassis for sustainable bioproduction. However, advanced design strategies for acetogens are currently hampered by incomplete knowledge about their physiology and our inability to accurately predict phenotypes. Here we describe the reconstruction of a novel genome-scale model of metabolism and macromolecular synthesis (ME-model) to gain new insights into the biology of the model acetogen *Clostridium ljungdahlii*. The model represents the first ME-model of a Gram-positive bacterium and captures all major central metabolic, amino acid, nucleotide, lipid, major cofactors, and vitamin synthesis pathways as well as pathways to synthesis RNA and protein molecules necessary to catalyze these reactions, thus significantly broadens the scope and predictability. Use of the model revealed how protein allocation and media composition influence metabolic pathways and energy conservation in acetogens and accurately predicted secretion of multiple fermentation products. Predicting overflow metabolism is of particular interest since it enables new design strategies, e.g. the formation of glycerol, a novel product for *C*. *ljungdahlii*, thus broadening the metabolic capability for this model microbe. Furthermore, prediction and experimental validation of changing secretion rates based on different metal availability opens the window into fermentation optimization and provides new knowledge about the proteome utilization and carbon flux in acetogens.

## Author summary

Acetogens are renowned for their potential biotechnological applications. The model acetogen *Clostridium ljungdahlii* has been studied intensively for its ability to produce biofuels from sustainable resources, like syngas. We describe a novel genome-scale model of *m*etabolism and gene *e*xpression (ME-model) to gain insights into this model acetogen. This first ME-model for a Gram-positive bacterium contains all major metabolic and biosynthetic pathways and calculates accurate proteome allocations under diverse growth conditions, thereby significantly broadening the scope of predictability of metabolic models. Furthermore, the ME-model enables rational medium design for improved production. Our experimental validation implies wide applicability to others strains for rapid improvement of yield and titer in biotechnology-relevant applications.

## Introduction

Acetogens have been investigated as promising alternative to convert waste gases containing CO_2_, H_2_, and CO (*i*.*e*., syngas) into multi-carbon commodities [1,2]. The Wood-Ljungdahl pathway (WLP) enables acetogens to use either H_2_ or CO as an electron donor with accompanied reduction of CO_2_, thereby making WLP the only known CO_2_-fixing pathway coupled to energy conservation [3]. Energetics of autotrophic growth was poorly understood for a long time as no ATP was gained at the substrate level, and not all acetogens contain cytochrome-encoding genes to maintain the proton motive force. It was recently discovered that proton exportation could be coupled to ferredoxin oxidation and NAD^+^ reduction by the Rnf complex [4]. Models like constraint-based genome-scale models of metabolism (*i*.*e*., M-models) have been useful for gaining insight to possible routes of energy flux [5–8]. While M-models have enabled much progress in elucidating cofactor fluxes, other critical components of the cell (*e*.*g*., production of macromolecules and mechanistic utilization of metals, vitamins, and cofactors) are usually absent in these models, thereby limiting in-depth understanding of cellular life.

So-called metabolic and gene expression models (ME-models) contain not only metabolic reactions, but represent all major cellular processes like macromolecular synthesis and basic transcriptional regulation, significantly broadening the scope and predictability of microbial systems biology [9,10]. In ME-models, both RNA and protein abundances are explicitly predicted, which means that cofactor requirements can now be explored. ME-models can compute the optimal molecular constitution of a cell as a function of genetic and environmental parameters, providing new inroads for advanced engineering designs.

Trace metals, fundamental for all living organisms, are required for catalytic processes essential to energy conservation, metabolism, replication, and maintenance. Yet metals pose a unique challenge for standard computational models as they are neither produced nor consumed biochemically in the model and are generally treated as a lumped sum in the biomass objective function [11], which prevents their proper integration into reactions [12]. ME-models change this paradigm because protein modifications are incorporated into these models. Protein modifications account for the presence of metals in biochemical reactions, thereby enabling predictions of optimal distribution of resources in response to limited metal availability. Thus, ME-models provide a robust, genome-wide approach to define how transition metals affect an organism’s functional network, which addresses the need to bridge chemistry and biology in a systematic way [12,13]. For acetogens, understanding the role of trace metals is particularly important, as metals are crucial for the WLP [14]. Insights into such requirements provide an opportunity to rationally manipulate the WLP and other pathways for improved biotechnological outcomes [15–17].

Here, we reconstructed and deployed the first ME-model of a Gram-positive bacterium. The completed *Clostridium ljungdahlii* ME-model, named iJL965-ME, captures all major central metabolic, amino acid, nucleotide, lipid, major cofactors, and vitamin synthesis pathways as well as pathways to synthesis RNA and protein molecules necessary to catalyze these reactions. Furthermore, the reconstruction includes WLP, with updated cofactors, and its associated mechanisms for energy conservation. The model accurately predicted secretion of acetate, ethanol, and glycerol during changing carbon and metal availability and revealed how protein allocation and media composition influence metabolic pathways and energy conservation in this model acetogen.

## Results

### Reconstructing an acetogen ME-model

**Fig 1:**
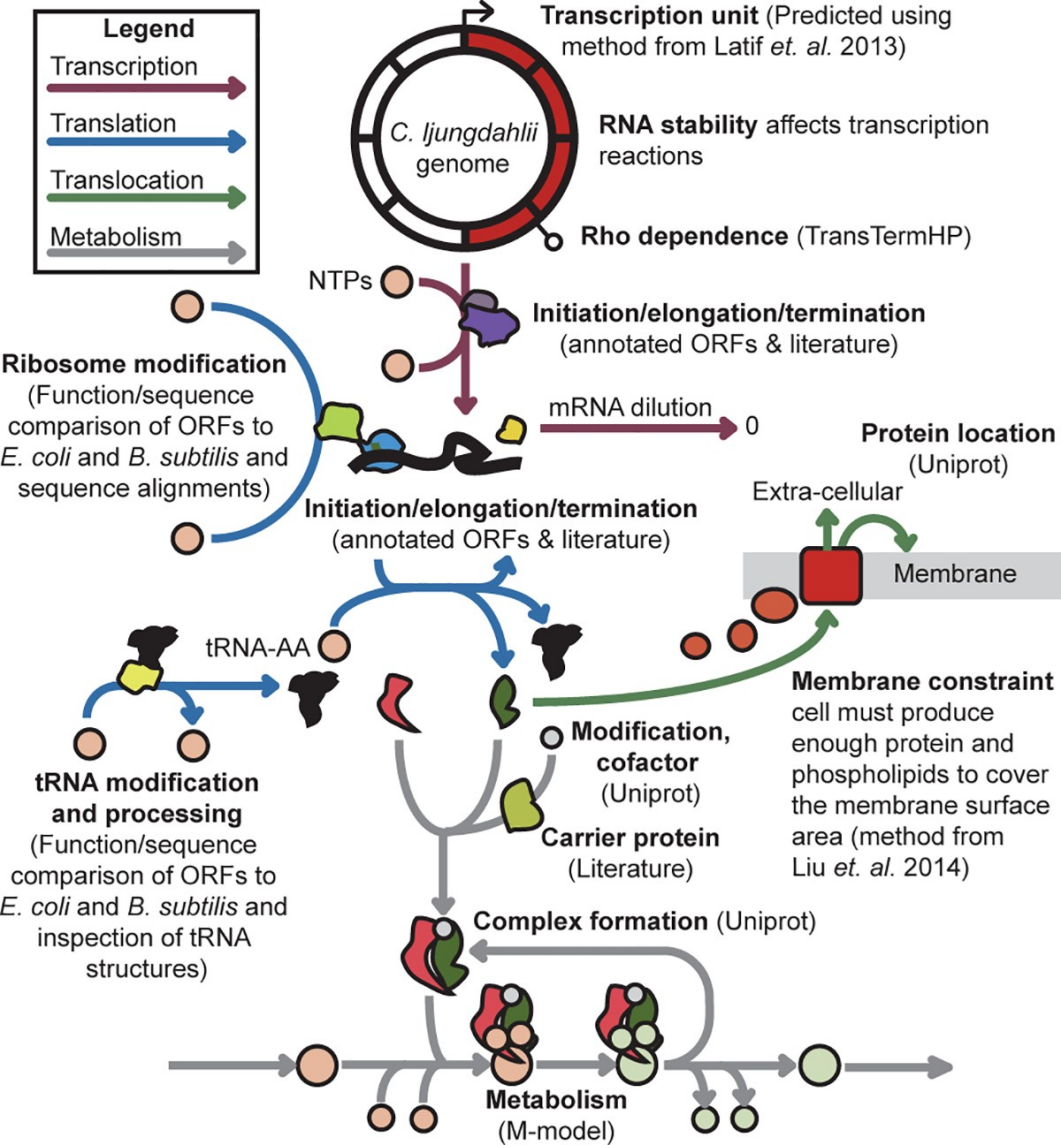
Representation of the ME-model. The E-matrix reconstruction accounted for transcription, translation, and translocation as well as associated reactions to produce functional enzymes. Integration of the E-matrix (colored arrows) with the M-model (grey arrows) resulted in the ME-model.

We first updated and created an existing genome-scale M-model of *C*. *ljungdahlii* (iHN637) [5]. By using recent literature and genome annotations as reference [18–22], 28 reactions were added and four reactions removed from iHN637. The updated M-model (iJL680) consisted of 43 additional genes (Supplemental file–iJL680.xml) and contained updated cofactor stoichiometry and directionality of redox reactions based on experimental data (Fig 1 in S1 File) and exhibits comparable predictability.

Next, a gene expression network (*i*.*e*., E-matrix) was reconstructed [23–26]. This reconstruction included an additional 196 protein-coding open reading frames (ORFs), 89 RNA genes, 576 transcription units (415 of which were rho-dependent and 29 were RNA-stable), 19 types of rRNA modifications, 17 types of tRNA modifications, 735 protein complexes with updated stoichiometry, 219 modified protein complexes, and 134 translocated proteins (Tables 1–15 in S2 File). Because accurate turnover rates for metabolic enzymes in *C*. *ljungdahlii* do not exist, this rate (approximated by *k*_*eff*_, a required parameter for ME-models) was set to 25 s^-1^, the average turnover rate of all enzymes in acetogens listed in Schiel-Bengelsdor and Dürre [1] and available on Brenda (accessed on Oct. 25, 2018) [27]. Coupling constraints, which link macromolecular synthesis costs with reactions, were calculated using the formulation in COBRAme [10,26,27].

Using the COBRAme framework, the acetogen E-matrix was integrated with iJL680 to create the ME-model (iJL965-ME; Supplemental file–iJL965_ME.pickle). iJL965-ME accounts for all of the major central metabolic pathways and biomass synthesis pathways as well as transcription, translation, macromolecule modifications, and translocation reactions (Fig 1). Because iJL965-ME covers an extensive scope of cellular processes, it enables prediction of fermentation profiles, including overflow metabolism products, gene expression, and usage of co-factors and metals, which are described in detail below.

### Accuracy of predicted growth and yield phenotypes improve with iJL965-ME

**Fig 2:**
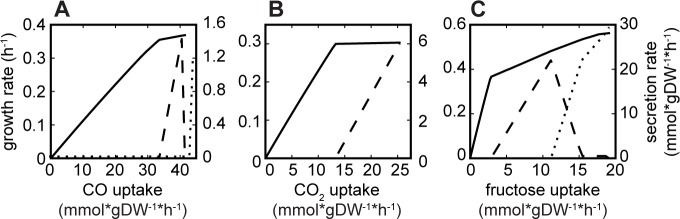
Predicted growth rate and yield. Maximum growth rate (solid line), acetate secretion rate (dashed line), and ethanol secretion rate (dotted line) changed as functions of (A) CO, (B) CO_2_, and (C) fructose uptake rate.

Unlike the M-model, iJL965-ME predicted both batch (*i*.*e*., maximum nutrient uptake) and nutrient-limited growth conditions. Due to internal constraints on protein production and catalysis, referred to as proteomic limitations [28], iJL965-ME growth rate was a non-linear function of the substrate uptake rate. Thus, optimal carbon uptake rate and maximum growth rate could be simultaneously predicted, whereas M-models require information of one rate to predict the other [10]. As a result, we identified unique growth rate and yield functions for growth with CO, CO_2_+H_2_, or fructose (Fig 2).

Overflow metabolism is the seemingly wasteful process in which a substrate is not fully oxidized, resulting in lower energy yields, inefficient metabolism, and additional fermentation products. Hypotheses for why this phenomenon occurs are varied, making characterization and modeling of mixed fermentation products challenging. Generally, M-models do not predict alternative fermentation products without additional constraints on redox fluxes, oxygen uptake, or the objective function [5–7,29]. However, iJL965-ME was able to predict intrinsically changes in the primary fermentation product as a function of substrate availability for CO and fructose growth. When protein production approached proteome limitations (exemplified by *in silico* maximum growth rate and *in vivo* mid-log phase), iJL965-ME correctly predicted the start of ethanol secretion after acetate secretion due to trade-offs in protein production (Fig 2A and 2C; Fig 2 in S1 File). Thus, iJL965-ME was able to recapitulate overflow metabolism by accounting for redox balancing and concurrent proteome limitations.

Although 2,3-butanediol has been described previously as potential secretion product, the model did not predict production of 2,3-butanediol because it promotes production of the most energy efficient metabolites (*i.e.*, ethanol and acetate). Furthermore, 2,3-butanediol is produced towards the stationary phase after acetate and ethanol [11], but the model assumes steady state growth for metabolic flux prediction. Therefore, we chose the exponential phase to measure the metabolites, since this is the best approximation to steady state and did not detect 2,3-butanediol.

**Fig 3:**
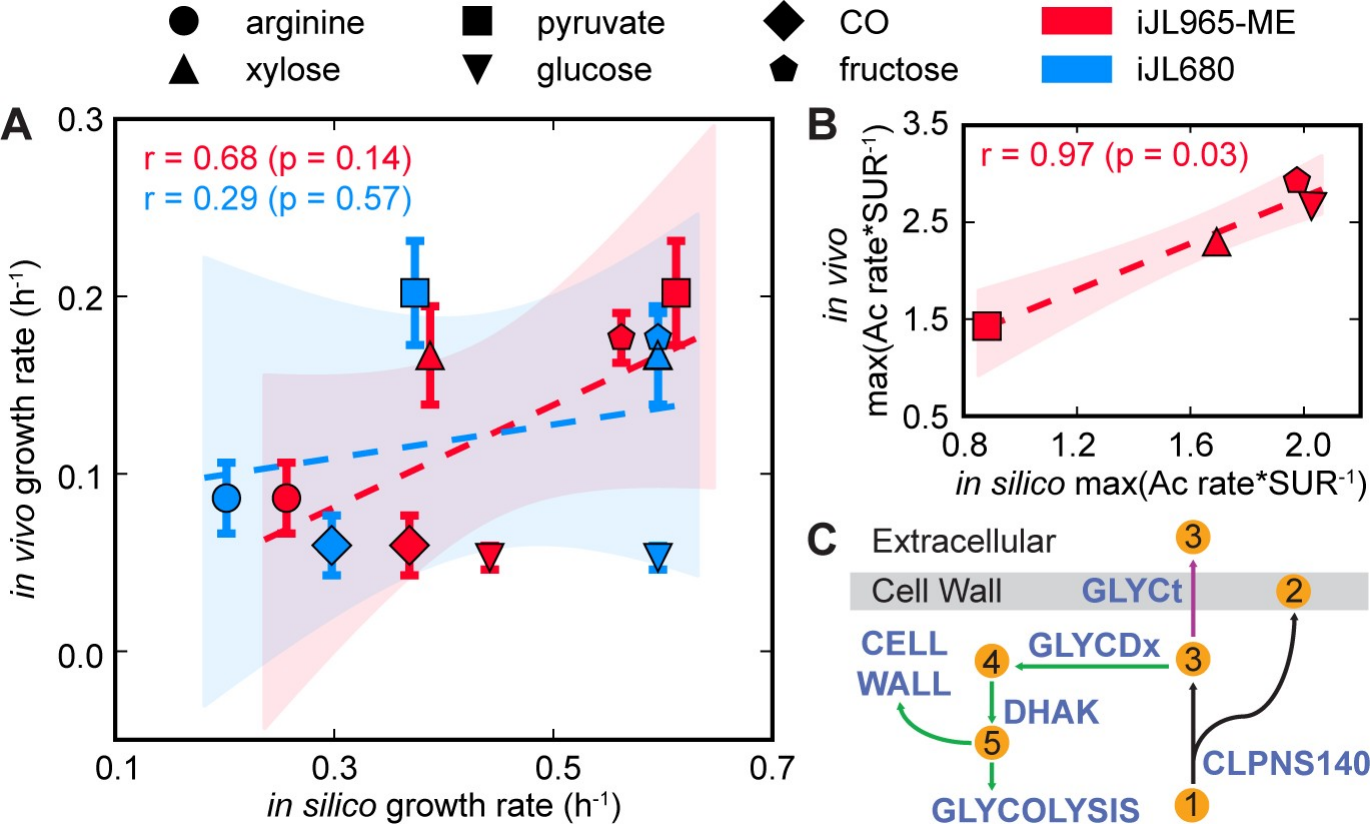
Predictions of growth rate and product production. (A**)** Two sets of predicted growth rates, from iJL680 and iJL965-ME, were plotted against *in vivo* measured growth rates for arginine, xylose, pyruvate, glucose, CO, and fructose growth conditions (±std, n = 3). Linear regressions and 95% confidence intervals were represented by dashed lines and shaded areas, respectively. In iJL680, carbon atom uptake was constrained to 30 mmol*gDW^-1^*h^-1^, while in iJL965-ME, the optimal carbon uptake was constrained by inherent proteome limitations. r and p represent Pearson's correlation and p-value. (B) Predicted maximum acetate secretion rate (Ac; mmol*gDW^-1^*h^-1^) to substrate uptake rate (SUR; mmol*gDW^-1^*h^-1^) was plotted against measured averaged values. (C) Predicted pathway mechanism for observed glycerol production in spent media. Glycerol was a byproduct of cell membrane formation during cardiolipin production. While the cell was carbon-limited, glycerol was recycled into biomass using the pathway highlighted in green. When cells were proteome-limited, *C*. *ljungdahlii* secreted glycerol (purple arrow). Abbreviations: 1 = phosphatidylglycerol (n-C14:0), 2 = cardiolipin (n-C14:0), 3 = glycerol, 4 = dihydroxyacetone, 5 = dihydroxyacetone phosphate, CLPNS140 = cardiolipin synthase (n-C14:0), GLYCt = glycerol transport, GLYCDx = glycerol dehydrogenase, DHAK = dihydroxyacetone kinase.

The ME-model also predicted substrate-specific growth rates with high accuracy. Specifically, growth rate predictions by iJL965-ME were more accurate than by M-model, iJL680 (Pearson’s r: 0.68 > 0.29; Spearman ρ: 0.60 > 0.091; Fig 3A). Due to distinct resource requirements (the main factor being proteome composition) when metabolizing different substrates, unique *in silico* maximum growth rates for individual substrates can be obtained through iJL965-ME. Unlike the M-model (iJL680), which predicted that glucose and fructose would have identical growth rates, iJL965-ME correctly predicted slower growth on glucose than for fructose. Furthermore, iJL965-ME highly improved predictions of the ratio of maximum acetate secretion rate to substrate uptake rate compared to the M-models iHN637 and iJL680 (Fig 3B; Table 16 in S2 File).

**Fig 4:**
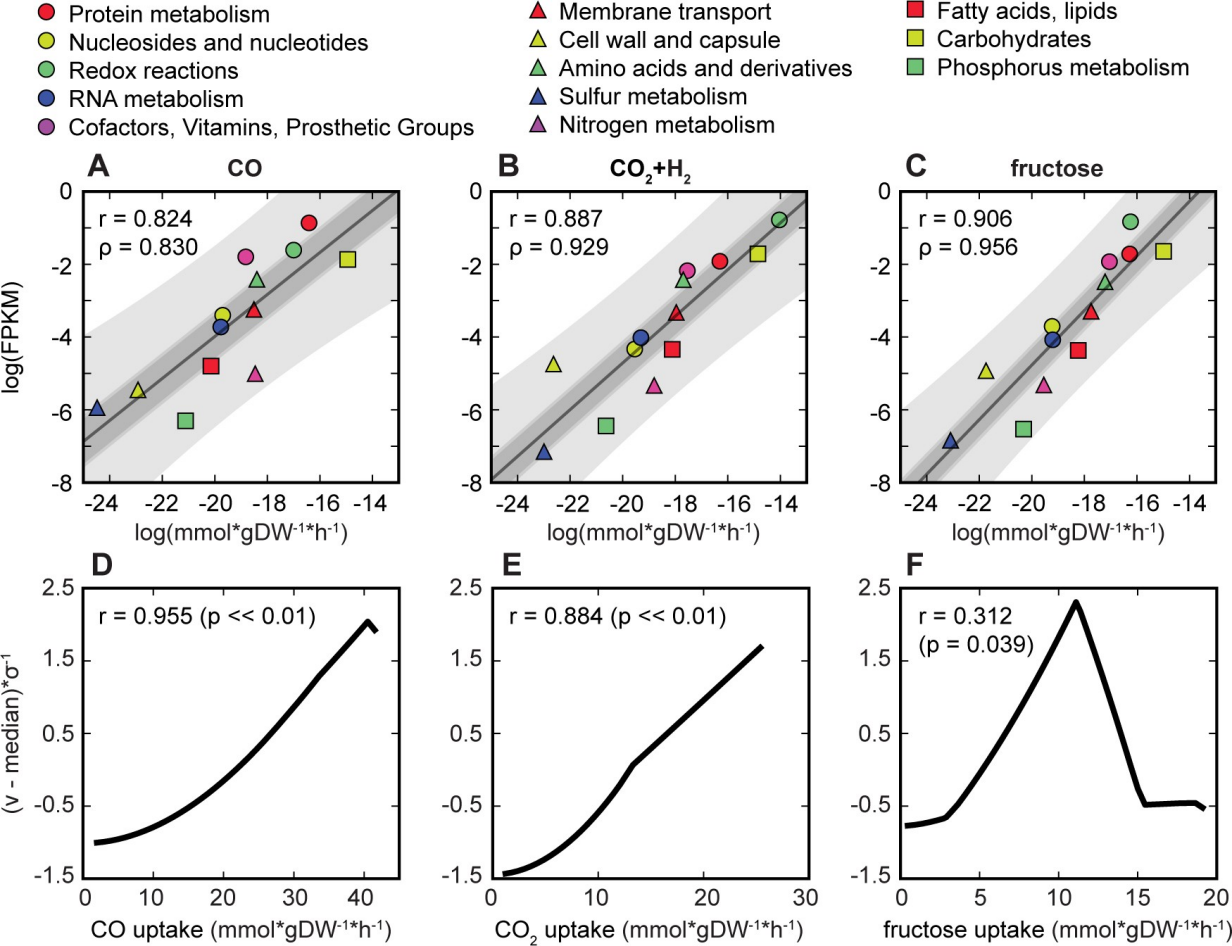
Predicted and experimental gene expression. Categorized by RAST subsystem and summed, predicted gene expression (transcription flux reactions * gDW of RNA molecule; mmol* h^-1^) was compared to RNA-seq data for *C*. *ljungdahlii* grown on (A) CO, (B) CO_2_+H_2_, and (C) fructose. Linear regressions, 95% confidence intervals of the regression, and 95% prediction intervals are represented by lines, dark shaded areas, and light shaded areas respectively. Scatter plots shown are for the highest Pearson r between predicted and experimental data. Normalized total transcription flux (mmol*gDW^-1^*h^-1^) of the Wood-Ljungdahl pathway was plotted against carbon substrate uptake rate for (D) CO, (E) CO_2_+H_2_, and (F) fructose. Pearson r reflects correlation with growth rate.

Interestingly, iJL965-ME predicted previously unknown secretion of glycerol (<2.5e^-3^ mmol*gDW^-1^*h^-1^) following acetate and ethanol production during growth on xylose or glucose but not on arginine, pyruvate, or CO, which may be due to where the substrate enters the metabolic network so that glycerol is produced through byproducts of glycolysis (xylose and glucose) or by reverse glycolysis (arginine, pyruvate, and CO). Like ethanol, glycerol secretion occurred due to trade-offs in proteome limitations resulting in overflow metabolism, as the cell no longer invested resources to recycle glycerol, a byproduct of cardiolipin production (Fig 3C). Glycerol production from cultures grown on either xylose or glucose was experimentally verified by high performance liquid chromatography (HPLC) analysis (0.024±0.012 mM and 0.083±0.018 mM glycerol for xylose or glucose, respectively; Fig 3 in S1 File), and was confirmed in glucose by gas chromatography/mass spectrometry (GC-MS) (Fig 4 in S1 File). Perhaps the levels of glycerol were too low to be detected in the xylose samples.

### Predicting gene expression

**Fig 5:**
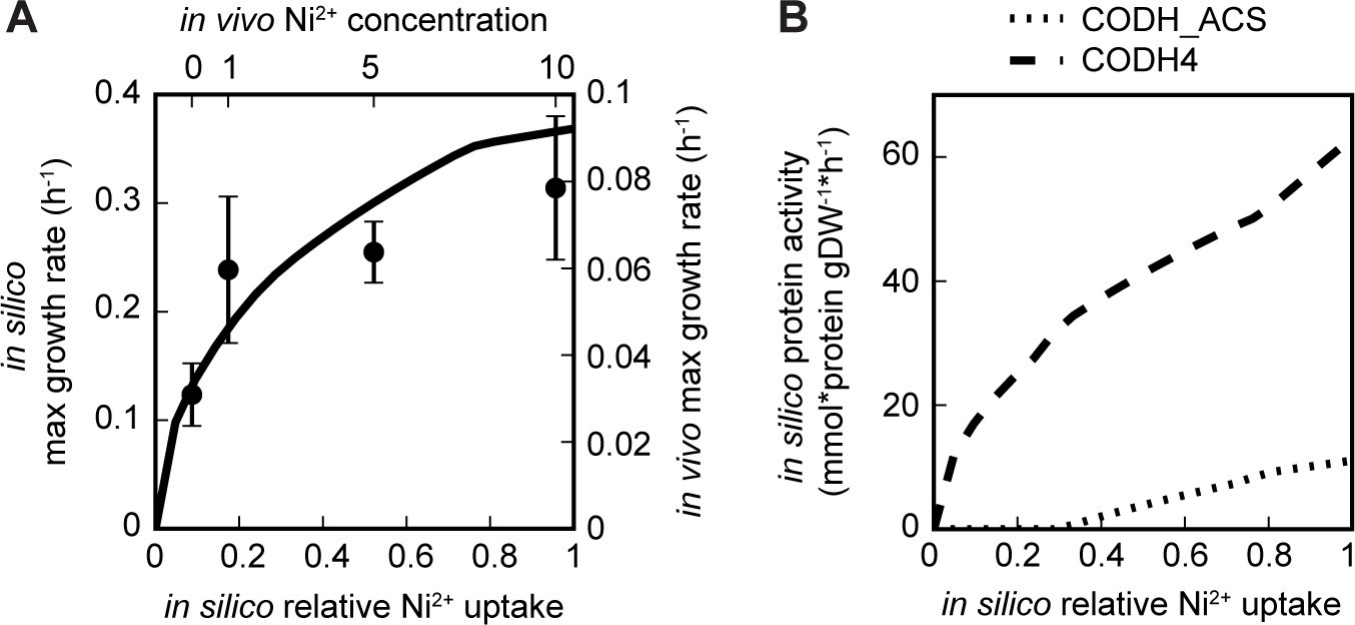
Effects of nickel availability on *C*. *ljungdahlii* grown on CO. (A) Maximum predicted growth rate was plotted against relative nickel uptake (line), and *in vivo* maximum growth rate verses the concentration of added nickel was plotted on the opposite axes (dot, ±std, n = 3). (B) Predicted protein activity of the nickel-containing enzymes, carbon monoxide dehydrogenase (CODH4) and carbon monoxide dehydrogenase:acetyl-CoA synthase (CODH_ACS), was plotted against relative nickel uptake.

**Fig 6:**
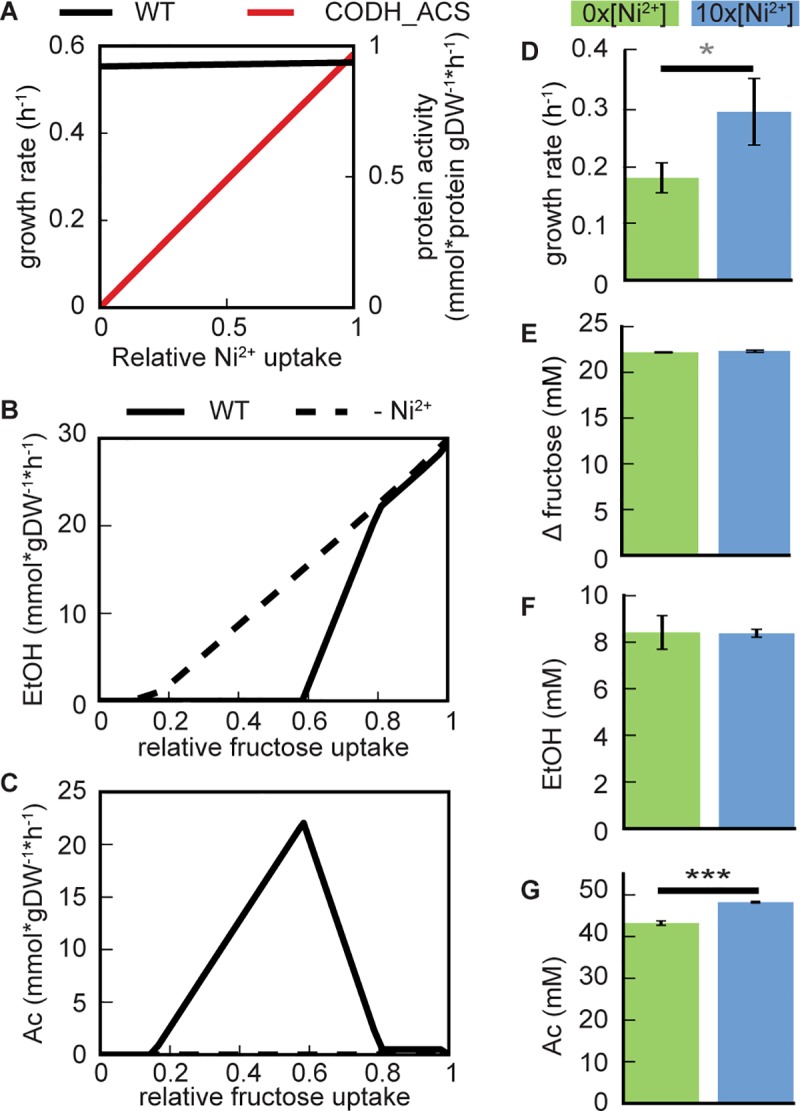
Effects of nickel availability on *C*. *ljungdahlii* grown on fructose. (A) Predicted growth rate and protein activity of carbon monoxide dehydrogenase:acetyl-CoA synthase (CODH_ACS) were plotted against relative nickel uptake (mmol*gDW^-1^*h^-1^). (B) Predicted ethanol (EtOH) secretion at optimal nickel uptake (WT) and no available nickel (-Ni^2+^) were plotted against relative fructose uptake (mmol*gDW^-1^*h^-1^). (C) Predicted acetate (Ac) secretion at optimal nickel uptake and no available nickel were plotted against relative fructose uptake (mmol*gDW^-1^*h^-1^). Measured (D) growth rate, (E) fructose consumption, (F) final ethanol concentration, and (G) final acetate concentration of fructose-grown *C*. *ljungdahlii* without added nickel and with ten times the concentration of nickel were plotted (±std, n = 3). Gray asterisk indicates difference significance is p = 0.06, and three black asterisk indicates significance of p<0.001.

RNA and protein abundance requirements are coupled to reaction fluxes in ME-models, enabling *in silico* predictions of transcription and translation (mmol*gDW^-1^*h^-1^) [10,26]. To test the accuracy of our model, genes were categorized by RAST subsystems and summed as per predicted transcription flux reactions (Table 17 in S2 File). The *in silico* results strongly correlated to RNA-seq data for *C*. *ljungdahlii* grown on CO, CO_2_+H_2_, or fructose (r > = 0.77, p< = 0.003; Fig 5 in S1 File) and to Ribo-seq data for *C*. *ljungdahlii* grown on CO or fructose (r> = 0.75, p< = 0.006; Fig 6 in S1 File) [30]. At the highest correlation, all categories fell within the prediction interval of the linear regression (Fig 4A–4C, Fig 7 in S1 File), enabling to forecast substrate-specific expression of pathways.

At the gene level, 396 genes could be strongly linked to growth rate (r>0.9, p value<0.05*Bonferonni, Fig 7 in S1 File). However, correlation of these genes was dependent on the growth substrate (68 genes for CO, 275 for CO_2_+H_2_, and 224 for fructose). Growth-correlated genes that were shared between conditions involved genes related to translation (*e.g.* rRNA and specific tRNAs; Fig 7 callout in S1 File).

Under autotrophic conditions, expression of WLP genes were correlated more with substrate availability than growth rate (r_CO_: 0.983>0.955, r_CO2+H2_: 0.996>0.884; Fig 4D and 4E). In addition, reaction fluxes of essential WLP reactions carbon monoxide dehydrogenase (CODH4) and 5,10-methylenetetrahydrofolate reductase (MTHFR5) were linearly related to CO uptake during growth on CO, while other non-WLP redox reactive reactions (*e*.*g*. RNF) were correlated with growth rate (Fig 8 in S1 File). Similarly, WLP reactions were linearly linked to CO_2_ uptake in CO_2_+H_2_ conditions, in addition to the linear response of ferredoxin:NADPH hydrogenase (HYDFDN2r) to H_2_, while non-WLP redox reactions were correlated with growth rate (Fig 9 in S1 File).

In heterotrophic conditions, the WLP was more active under nutrient limitations than proteome limitations, as its activity level was related to acetate secretion (r = 0.993, p<0.01, Fig 4F). The WLP was recapturing CO_2_ for biomass production using the reducing power gained by metabolizing fructose. At greater than 57% of the optimal fructose uptake (Fig 4F), the primary provider of oxidized ferredoxin switched from WLP to ferredoxin:NADP reductase, also known as the Nfn complex (FRNDPR2r) and acetaldehyde:ferredoxin oxidoreductase (AOR_CL) (Fig 10 in S1 File). Extraneous reducing power captured by NAD^+^ from glyceraldehyde-3-phosphate dehydrogenase (GAPD) was removed by producing ethanol (alcohol dehydrogenase; ALCD2x) (Fig 10 in S1 File). These findings are corroborated by a previous report that *C*. *ljungdahlii* grows mixotrophically, instead of heterotrophically, when presented with sugar as a carbon source [31].

### Nickel controls phenotype through Wood-Ljungdahl activity

Metal availability and growth rate are linearly correlated in M-models, even though there is contrary experimental evidence [32]. For example, seven of ten metals (Ca^2+^, Cu^2+^, Mg^2+^, Mn^2+^, Mo^2+^, Ni^2+^, Zn^2+^ + Co^2+^, Fe^2+^, Na^+^) could only be imported or exported, and only Co was predicted to participate in flux-carrying reactions that were not a transport reaction or biomass production [5]. Thus, most metal ions are not associated to the reactions they help catalyze.

Cofactor integration in iJL965-ME, however, allows systematic interrogation of the effects of metal availability. Particularly, iJL965-ME’s nickel-containing proteins, CODH4 and carbon monoxide dehydrogenase:Acetyl-CoA synthase (CODH_ACS), are part of the WLP, while a third nickel-containing protein (a hydrogenase, HYD2) does not carry flux on CO. This network configuration afforded the possibility of controlling this pathway through changes in media composition both *in silico* and *in vivo*. Due to *C*. *ljungdahlii*’s reliance on WLP for autotrophic growth, nickel was predicted to be essential for CO-growth, which was experimentally confirmed in the related acetogen *C*. *ragsdalei* [32]. Although true essentiality could not be tested due to trace nickel contamination in the media, the amount of additional nickel (added as multiples of 0.10 mM) significantly influenced *in vivo* growth rate in a quadratic fashion as predicted (Fig 5A) and previously demonstrated in *C*. *ragsdalei* [32]. Experiments with *C*. *ragsdalei* also showed that nickel availability affected the specific activity of carbon hydroxide dehydrogenase (CODH) [32]. According to iJL965-ME, the non-linear effects of nickel limitations were caused by an uneven distribution of metal resources between CODH_ACS and CODH4, resulting in different rates of decreasing protein activity (Fig 5B). In turn, the other reactions in WLP were correlated to either CODH_ACS, like MTHFR5 and methyltetrahydrofolate corrinoid/iron-sulfur protein methyltransferase (METR), or CODH4 (Fig 11 in S1 File). Finally, iJL965-ME predicted that while nickel availability affected growth rate, protein activity, and acetate and ethanol yield, the acetate:ethanol production rate would not change, instead it remained constant at 1.4 for different nickel concentrations (Fig 12A in S1 File). Indeed, acetate:ethanol production rate, as determined by HPLC, was unchanged with a ratio of 1.48±0.34 (Fig 12E in S1 File), regardless of the nickel concentrations used (0x, 1x, and 5x [10x excluded due to carbon depletion], Fig 12D in S1 File).

iJL965-ME predicted that nickel limitations would have different effects on fructose-grown cells. Removal of nickel was not predicted to affect growth rate or fructose uptake significantly (Δ_gr_ = 98%, Δ_fructose_ = 99%, Fig 6A). However, there was no CODH_ACS or METR activity under nickel depletion, which reduced the WLP activity (Table 18 in S2 File) and eliminated acetate secretion. Instead, the model predicted that only ethanol secretion would occur (Fig 6B and 6C). To test this prediction, *C*. *ljungdahlii* was grown either without added nickel (0x) or with high nickel concentrations (10x). Both cultures consumed the same amount of fructose (p = 0.26) and produced identical amounts of ethanol (p = 0.95), but exhibited different growth rates (p = 0.062) and final concentrations of acetate (p = 2.2e^-4^) (Fig 6D–6G). Increased acetate secretion rate (p = 0.016, Fig 13 in S1 File) and final acetate concentrations in 10x nickel were due to the nickel-stimulated WLP consuming more CO_2_.

## Discussion

We showed that the incorporation of the E-matrix into constraint-based genome-scale models significantly widens the scope of their application, including prediction of overflow metabolism and optimal expression levels, as well as media optimization strategies. Such capabilities proved useful for exploring and understanding system responses of *C*. *ljungdahlii*. The reconstructed *C*. *ljungdahlii* ME-model (iJL965-ME) was not only more accurate than the M-model at predicting growth rates and acetate secretion rates, but was also capable of predicting secretion of ethanol and until now unknown secretion of glycerol (Figs 2 and 3). Furthermore, *in silico* predictions of gene/subsystem expression were highly comparable to *in vivo* transcriptomics for three separate conditions, bolstering confidence in predicting macromolecular responses to environmental changes (Fig 4A–4C). C1 metabolism under both autotrophic and mixotrophic conditions was examined in more depth, and the potential of controlling WLP activity through media composition was explored (Figs 4–6). Although the lack of CODH_ACS activity (achieved by removing nickel from the media) may not cease WLP activity entirely, it may stop acetate production (as *in vivo* nickel depletion results suggest), leading to ethanol production as the main fermentation end product (Fig 6). However, the discrepancy between *in silico* and *in vivo* growth rates of nickel-depleted cells grown on fructose implied that WLP was more important than predicted for maximizing growth in mixotrophic conditions (Fig 6) and could be due to regulatory effects. In contrast, nickel was essential for CO-growth, but had no effect on the acetate:ethanol ratio (Fig 5).

ME-models provide a comprehensive, genome-scale, systems biology approach to link the environment with macronutrient metabolism. In particular, the combination of C1 metabolism, multi-omics predictions, and cofactor integration into iJL965-ME is an important milestone for a holistic understanding of metals in metabolism. Although nickel was the only trace metal to be investigated here, iJL965-ME invites further studies elucidating specific effects of concurrent metal limitations and genetic perturbations. The ME-model represents an inclusive method that unites analysis and integration of multiple data types.

## Materials and methods

### Bacterial growth conditions

*Clostridium ljungdahlii* (ATCC 55383) was grown under anaerobic conditions containing PETC medium (ATCC medium 1754 without fructose) at 37°C. Fructose cultures were grown in 125 mL serum bottles containing 100 mL of medium plus 28 mM fructose, CO in 125 mL serum bottles containing 25 mL of media and bottles were pressurized once with CO to 1.25 bar. Pyruvate, xylose, glucose, and arginine experiments were performed in test tubes containing 10 mL of medium and equimolar concentrations of carbon atoms (30) per carbon source, such as 5mM fructose and 10mM pyruvate. Medium contained 0.10 mM of NiCl_2_*6 H_2_O (defined as 1x). For testing the effect of nickel, final concentrations of 0 mM (0x), 0.50 mM (5x) and 1.0 mM (10x) of nickel were added to the media before autoclaving. Growth was routinely determined by measurement of OD_600_. Concentrations of fructose, acetate, ethanol, and glycerol were determined by high-performance liquid chromatography (Waters) as previously described [33]. Detection was performed by UV absorption at 410 nm.

### GC-MS for glycerol detection

The presence of glycerol in the cell cultures samples was investigated with GC-MS. An Agilent 7890B GC with a 7200 Accurate Mass QTOF MS (Agilent, Santa Clara, CA) with an Electron Ionization source (EI) instrument was used. GC separation was carried out on a HP-5ms (5%-Phenyl)-methylpolysiloxane GC column (Agilent, Santa Clara, CA) with ID of 0.25 mm, 30 m length and 0.25 μm film thickness. Prior to analysis, the 0.2 ml sample aliquot was lyophilized at room temperature and reconstituted in 50 μL of HPLC-grade methanol with 5 sonication. A 20 μL aliquot of supernatant was then carefully transferred into a 2 mL vial with a spring insert and capped with a septum cap.

For analysis, a 1 μL aliquot of sample was injected by the auto-sampling robot. The GC inlet was maintained at 250°C and set for 10:1 split. The GC separation was as follows: start at 40°C and hold for 1 min; 20°C/min oven ramp to 45°C, hold of 0.1 min; 20°C/min oven ramp to 300°C, hold for 0.1 min; 50°C/min oven ramp to 320°C for a complete run time of 14.6 min. The helium carrier gas was set to constant 1.2 mL/min flow. The scanned *m/z* range of TOF MS analyzer was set to 35–400 amu with acquisition rate of 20 spectra/second. For the first 1.65 min of the analysis the detector was turned off (solvent delay). The methanol solvent blanks and empty vial blanks were interspersed with the samples; a solvent blank was run prior to each sample to ensure absence of carryover. In order to eliminate potential systematic bias, the samples were randomized.

The chromatograms were analyzed using Agilent’s MassHunter software v. B.08.00. Prior to analysis, an authentic standard of glycerol at ~1 mM concentration processed in the same fashion as the samples, was injected to determine the retention time of the compound for the analysis conditions and establish that the EI fragmentation pattern obtained on the instrument is identical to that in the search library (Wiley Registry of Mass Spectral Data, 11^th^ Edition). The library matching was performed using the NISM MS Search software v. 2.3.

### RNA isolation, removal of rRNA and library preparation of CO-grown cells

All experiments were performed using two biological replicates. Cell pellets were collected by centrifugation at room temperature for 5 mins at 5000 g. Growth medium was removed and cell pellets were snap frozen immediately in liquid nitrogen, then kept at -80°C. Cell lysates were prepared by grinding the pellets in liquid nitrogen. The lysates were cleared by centrifugation (13000 g) at 4°C. To stabilize RNA, 500 μl of Trizole reagent (Thermo Fisher Scientific) was added to 50–100 μl of cleared cell lysates, vortex mixed and stored at -80°C. The samples were brought to room temperature and 140 μl of chloroform was added to each tube, vortex mixed and centrifuged at maximum speed at 4°C for 10 mins. The aqueous fraction was isolated and total RNA was extracted using the RNeasy mini kit (Qiagen), the volume was brought to 900 μl using RLT buffer, 600 μl of 95% ethanol was added and mixed in order to bind the RNA. The RNeasy protocol was then followed as recommended by the manufacturer to isolate pure RNA. The ribosomal RNA (rRNA) was depleted using the Ribo-Zero rRNA Removal kit (Illumina). Strand-specific RNA-seq libraries were prepared using the Stranded RNA-seq Kit (Kapa Biosystems). The libraries were paired-end sequenced with Illumina HiSeq 4000. The sequencing reads were mapped to the *C*. *ljungdahlii* genome NC_014328 with Bowtie2. FeatureCounts was used to estimate reads per gene. DESeq2 was used to determine differentially expressed genes. RNA-seq values were FPKM-normalized. Reads were deposited to BioSample as SAMN07391098.

### Revision of M-model

A previously published M-model, iHN637, was updated to remove obsolete metabolic reactions and add new reactions to reflect current literature [18,19,24]. The *C*. *ljungdahlii* genome was reannotated using RAST and PROKKA to account for the most recent information and methods in functional annotations [20,22]. If both start and end sites of ORFs matched that of the original annotation but the functions did not, the new function was also considered during reconstruction of both M- and ME-models. Flux Balance Analysis simulations [34] were carried out as described previously using COBRApy [35]. All M-model simulations maximized growth through the biomass objective function [36].

### Reconstructing the ME-model

Bidirectional hits and functional overlaps (using RAST annotations) between *Escherichia coli*, *Bacilllus subtilis*, and *C*. *ljungdahlii*, as well as manual curation of the published annotation, and genome annotations obtained by RAST and PROKKA were used to identify potential E-matrix proteins [20–22]. Using *E*. *coli* [9,10,25] and *B*. *subtilis* [22] as reference and the method established by Thiele *et al*. to fill in missing knowledge, template reactions [25] for the following functions were reconstructed: essential rRNA and tRNA modifications, transcription, translation, translocation, a single bilayer membrane constraint, and Fe-S cluster formation. Transcription units were downloaded from MetaCyc on March 23, 2015 [37] and rho independent TUs were predicted using ARNold [38]. The gene-protein-reactions in iHN673 were converted into protein complexes and updated using Uniprot and PDB annotations as well as functional similarity to *E*. *coli* and *B*. *subtilis* proteins. The modeled protein complexes contained updated stoichiometry and modifications. COBRAme was used to comprise this information into a cohesive model [26].

All parameters from COBRAme were carried over except for the following: The (non) growth associated maintenance (instead set to iHN637’s); the unmodeled protein proportion of proteome (set to 0.35 based on relative protein weight of unmodeled proteins using RNA-seq data as a 1-to-1 proxy for protein levels); and median enzyme efficiency (set to 25/s, based on the average turnover rate of all acetogen enzymes listed in Schiel-Bengelsdorf and Dürre [1] and downloaded from Brenda [27] on 10/25/15). CLJU_c00670 was used as the “unmodeled protein”, as it was the highest expressed unmodeled protein in CO_2_+H_2_ and fructose RNA-seq data [5]. COBRAme also requires a “dummy protein” to solve for max growth rate [26]. In iJL965-ME, this was a 26 amino acid protein used to catalyze reactions that required an enzyme catalyst but did not have an identified homologue in *C*. *ljungdahlii*. For example, a membrane version of the protein was used to transport metabolites without an assigned transporter. Demands for glycerol, DNA, murein, ATP maintenance were added based on the iHN637 biomass objective function [5]. Metabolic coupling constraints were added to ferredoxin and thioredoxin when they get reduced; otherwise, they would not be expressed despite their importance.

tRNA modifying proteins were identified from the genome annotation. Secondary structure of tRNAs were predicted using tRNAscan-SE [39]. Each tRNA was manually checked to see if they met the requirements for modification. If yes, then their modifying reactions were added to the model (Tables 7–9 in S2 File). Selenocysteine was not included.

Similarly, ribosome-modifying proteins were identified from the genome annotation. These proteins were then checked in literature to see if they were essential for *E*. *coli* or *B*. *subtilis*. Then, target sequences for modification were identified to see if they existed in *C*. *ljungdahlii*. If both requirements existed, their modifying reactions were added to the model (Tables 4–6 in S2 File).

NifU was used as the carrier protein for iron-sulfur formation, while sulfur was added using IscS as the sulfur carrier and SufBC as the catalyst [40]. ErpA was used for final step of iron-sulfur transfer. Hyp was used for nickel transfer [40].

### Analyzing the ME-model

Using SoPlex and cobrapy, growth rate was optimized using binary_search(), as described in COBRAme [26,35,41]. All analysis was carried out using python scripts and python in Jupyter Notebooks, and visualization was provided by matplotlib [42,43]. An example Jupyter Notebook containing code that can recreate Fig 5 is included in the supplements. Scipy and statsmodels were used for statistical analysis [44,45]. All error bars were 1 standard deviation. In comparing *in vivo* data to *in silico* data, RNA-seq and Ribo-seq reads from *C*. *ljungdahlii* grown on fructose, CO_2_+H_2_, and CO that correspond to the 965 modeled ORFs were summed and logged [5,30]. To calculate the p-values, the expression values of the 965 ORFs were randomly shuffled and the Pearson r values recalculated 1000 times.

The model used in this paper is provided as a pickle file (iJL965_ME.pickle). COBRAme-compatible versions are available as json files. A version containing all of the reactions and metabolites in iJL695-ME is available as iJL965_ME_reduced.json, and a fully functioning json version can be created by loading iJL965_ME_full.json and running load_iJL965_me.

## Supporting information

S1 File Document containing supplemental figures. (DOCX)

S2 File Spreadsheet document containing supplemental tables. (XLSX)

S3 File An .xml file that contains a *C*. *ljundahlii* metabolic model with 680 genes. (XML)

S4 File A python pickle file that contains a *C*. *ljungdahlii* metabolic and gene expression model with 965 genes. (PICKLE)

S5 File A json file that contains a reduced and COBRAme-compatible version of iJL965-ME. (JSON)

S6 File A Jupyter notebook that contains code that can be used to load iJL965-ME, solve the model, and reproduce a version of Fig 5. (IPYNB)

S7 File A zipped folder containing the json files and python scripts to get a COBRAme-compatible version of iJL965-ME. (ZIP)
