# Supplementary material for: Predicting proteome allocation, overflow metabolism, and metal requirements in a model acetogen
Source: PLoS Comput Biol. 2019 Mar 7;15(3):e1006848. doi: 10.1371/journal.pcbi.1006848 (PMC6430413; doi:10.1371/journal.pcbi.1006848)
Supplement: S1 File — (DOCX) [file pcbi.1006848.s001.docx]

**
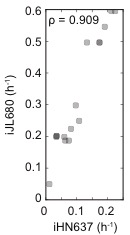
S1 Figure. Comparing predicted growth rates of iHN637 to iJL680, the updated *C. ljungdahlii* M-model.** Queried substrates and uptake rates are the same from table 1 in Nagarajan, H. *et al.* [1].

**S2 Figure. Measured growth curves, substrate uptake, and products for autotrophic and heterotrophic conditions.** Growth curves (OD_600_, black line) and HPLC measured molecules were plotted against hours for **a,** CO and **b,** fructose as carbon source.

**S3 Figure. Predicted and measured growth curves, substrate uptake rates, and yield.** Predicted growth rate (black line; h^-1^) and secreted products (acetate, ethanol, succinate, glycerol; mmol*gDW^-1^*h^-1^) were plotted against substrate uptake rate (mmol*gDW^-1^*h^-1^) in the left column. Growth curves (OD_600_, black line) and HPLC measured molecules were plotted against hours in the middle column. Rates (mmol*gDW^-1^*h^-1^) were calculated from the middle column using the first derivative of a Savitzky-Golay filter, and products were plotted against carbon uptake rate. Carbon source per row was as follows: **a** & **b,** arginine, **c-e,** glucose, **f-h,** pyruvate, and **i-k,** xylose.

**S4 Figure. Detection of glycerol with GC-MS.** **a,** An example fragment of gas chromatograms overlaid for several samples; the marked retention time corresponds to that determined for the standard of glycerol under the utilized protocol (~5.4 min). High abundance is detected in glucose t_241_, low abundance in glucose t_226_, and no glycerol was detectable in other samples as well as media and solvent blanks. **b,** Mirror plot of the experimental fragmentation pattern (top, red) and the library search spectrum (bottom, blue) confirming the identity of glycerol. Both forward and reverse search score were above 800 which indicate high confidence of identification [2].

**S5 Figure. Pearson r correlation between categorized and summed predicted transcriptomics.** The Pearson r correlation between categorized and summed predicted transcription flux reactions and RNA-seq data was calculated for discrete substrate uptake rates that ranged from maximum to low uptake rates (n>30, 0 unfeasible). Relative substrate uptake rate for CO, CO_2_, and fructose was plotted against the Pearson r.


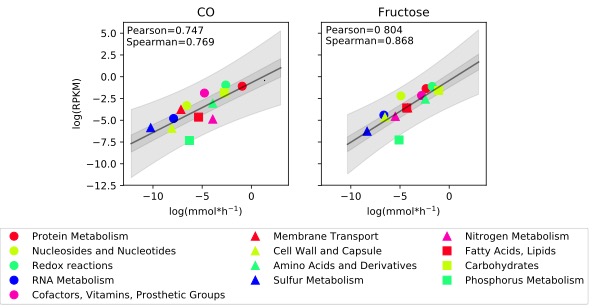


**S6 Figure. Predicted and experimental protein expression.** Categorized by RAST subsystem and summed, predicted protein expression (translation flux reactions * protein weight based on amino acid composition) was compared to Ribo-seq data for *C. ljungdahlii* grown on CO and fructose (from Al-Bassam *et al.* [3]). Linear regressions, 95% confidence intervals of the regression, and 95% prediction intervals are represented by lines, dark shaded areas, and light shaded areas respectively. Scatter plots shown are for the highest Pearson r identified in Figure 4.


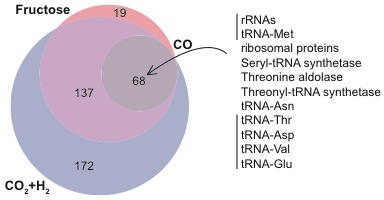


**S7 Figure. Genes highly correlated with growth rate.** Genes that were highly correlated with growth rate more so than substrate uptake rate (r > 0.9, p-val < 0.05*Bonferonni) were identified, and overlap of genes between the three substrate conditions were plotted in a venn diagram. In the call-out of gene functions shared in all three conditions, the black vertical line indicates that these genes were in the same operon.

**
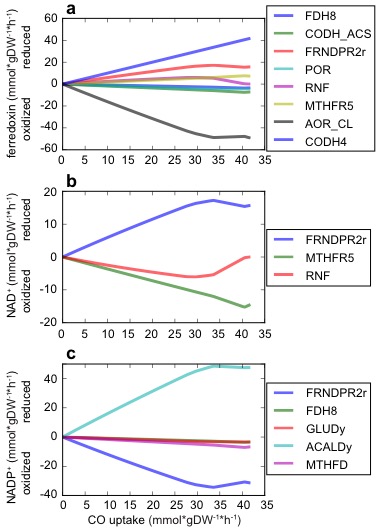
S8 Figure. Predicted high flux-carrying redox reactions on CO-growth.** Fluxes were plotted against CO uptake rate for reactions involving **a,** ferredoxin, **b,** NAD^+^, and **c,** NADP^+^. High flux was defined as the absolute sum of flux across the nutrient spectrum greater than 40 mmol*gDW^-1^*h^-1^. Abbreviations: FDH8 = ferredoxin dehydrogenase, CODH_ACS = carbon monoxide dehydrogenase, FRNDPR2r = ferredoxin:NADP reductase, POR = pyruvate synthase, RNF = ferredoxin:NAD oxidoreductase, MTHFR5 = 5,10-methylenetetrahydrofolate reductase (ferredoxin), AOR_CL = acetaldehyde:ferredoxin oxidoreductase, CODH4 = carbon monoxide dehydrogenase, GLUDy = glutamate dehydrogenase (NADP), ACALDy = acetaldehyde dehydrogenase, MTHFD = methylenetetrahydrofolate dehydrogenase (NADP).

**
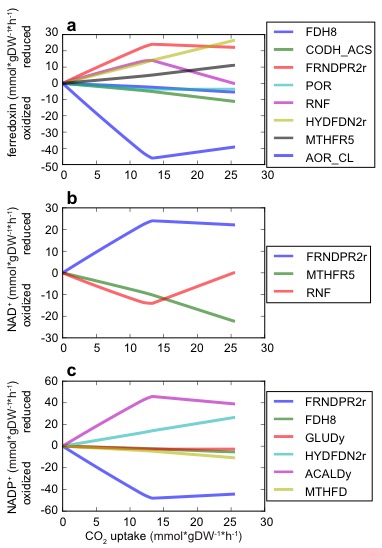
S9 Figure. Predicted high flux-carrying redox reactions on CO2-growth.** Fluxes were plotted against CO_2_ uptake rate for reactions involving **a,** ferredoxin, **b,** NAD^+^, and **c,** NADP^+^. High flux was defined as the absolute sum of flux across the nutrient spectrum greater than 40 mmol*gDW^-1^*h^-1^. Abbreviations: FDH8 = ferredoxin dehydrogenase, CODH_ACS = carbon monoxide dehydrogenase, FRNDPR2r = ferredoxin:NADP reductase, POR = pyruvate synthase, RNF = ferredoxin:NAD oxidoreductase, MTHFR5 = 5,10-methylenetetrahydrofolate reductase (ferredoxin), AOR_CL = acetaldehyde:ferredoxin oxidoreductase, GLUDy = glutamate dehydrogenase (NADP), ACALDy = acetaldehyde dehydrogenase, MTHFD = methylenetetrahydrofolate dehydrogenase (NADP), HYDFDN2r = ferredoxin:NADPH hydrogenase.

**
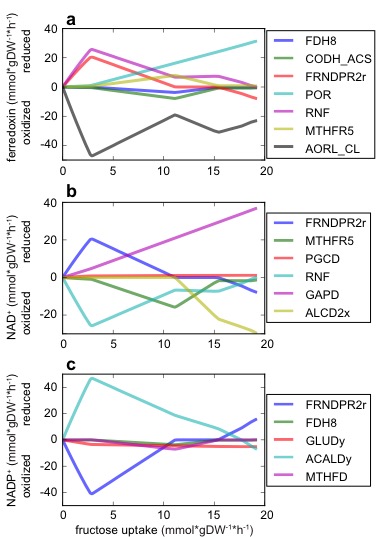
S10 Figure. Predicted high flux-carrying redox reactions on fructose-growth.** Fluxes were plotted against fructose uptake rate for reactions involving **a,** ferredoxin, **b,** NAD^+^, and **c,** NADP^+^. High flux was defined as the absolute sum of flux across the nutrient spectrum greater than 40 mmol*gDW^-1^*h^-1^. Abbreviations: FDH8 = ferredoxin dehydrogenase, CODH_ACS = carbon monoxide dehydrogenase, FRNDPR2r = ferredoxin:NADP reductase, POR = pyruvate synthase, RNF = ferredoxin:NAD oxidoreductase, MTHFR5 = 5,10-methylenetetrahydrofolate reductase (ferredoxin), AOR_CL = acetaldehyde:ferredoxin oxidoreductase, GLUDy = glutamate dehydrogenase (NADP), ACALDy = acetaldehyde dehydrogenase, MTHFD = methylenetetrahydrofolate dehydrogenase (NADP), GAPD = glyceraldehyde-3-phosphate dehydrogenase, PGCD = phosphoglycerate dehydrogenase.

**S11 Figure. Heatmap of Pearson correlations of WLP reaction fluxes over nickel availability of CO-grown cells and clustered by Euclidean distance.** Abbreviations: MTHFR5 = 5,10-methylenetetrahydrofolate reductase (ferredoxin), METR = methyltetrahydrofolate corrinoid/iron-sulfur protein methyltransferase, CODH_ACS = carbon monoxide dehydrogenase, FDH8 = ferredoxin dehydrogenase, CODH4 = carbon monoxide dehydrogenase, MTHFC = methenyltetrahydrofolate cyclohydrolase, MTHFB = methylentetrahydrofolate dehydrogenase.

**S12 Figure. Predicted and measured acetate and ethanol secretion rates of CO-grown *C. ljungdahlii* with varying nickel availability.** **a,** Predicted maximum acetate and maximum ethanol secretion rates as well as the acetate-to-ethanol ratio were plotted against relative maximum constrained nickel uptake. Bar graphs of measured **b,** maximum ethanol secretion rates, **c,** maximum acetate secretion rates, **d,** total carbon from final concentration of acetate and ethanol at t = 116 hours, and **e,** acetate-to-ethanol ratio were plotted for 4 different concentrations of added nickel (±std, n=3). Horizontal lines indicate significant differences.

**
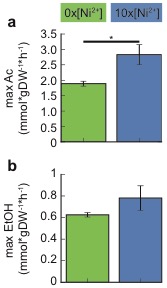
S13 Figure. Measured secretion rates of acetate and ethanol of fructose-grown *C. ljungdahlii* with and without nickel.** Bar graphs of measured **a,** maximum acetate (Ac) secretion rates and **b,** maximum ethanol (EtOH) secretion rates of fructose-grown *C. ljungdahlii* without added nickel (0x) and with concentrated nickel (10x) (±std, n=3). Black asterisk indicates significance of p<0.05.

**Reference**

1. Nagarajan H, Sahin M, Nogales J, Latif H, Lovley DR, Ebrahim A, et al. Characterizing acetogenic metabolism using a genome-scale metabolic reconstruction of *Clostridium ljungdahlii*. Microb Cell Fact. 2013;12: 118. doi:10.1186/1475-2859-12-118

2. Stein S. Mass spectral reference libraries: an ever-expanding resource for chemical identification. Anal Chem. 2012;84: 7274–7282.

3. Al-Bassam MM, Kim J-N, Zaramela LS, Kellman BP, Zuniga C, Wozniak JM, et al. Optimization of carbon and energy utilization through differential translational efficiency. Nat Commun. 2018;9: 4474.
